# Supplementary material for: Evaluation of Intradermal PRRSV MLV Vaccination of Suckling Piglets on Health and Performance Parameters under Field Conditions
Source: Animals (Basel). 2022 Dec 23;13(1):61. doi: 10.3390/ani13010061 (PMC9817773; doi:10.3390/ani13010061)

A) ELISA - qRT-PCR

F tests - ANOVA: Repeated measures, within-between interaction

Analysis: A priori: Compute required sample size

Input: Effect size f = 0.5

α err prob = 0.05

Power (1-β err prob) = 0.95

Number of groups = 4

Number of measurements = 6

Corr among rep measures = 0.2

Nonsphericity correction ε = 1

Output: Noncentrality parameter λ = 37.5000000

Critical F = 1.7932218

Numerator df = 15.0000000

Denominator df = 80.0000000

Total sample size = 20

Actual power = 0.9773699

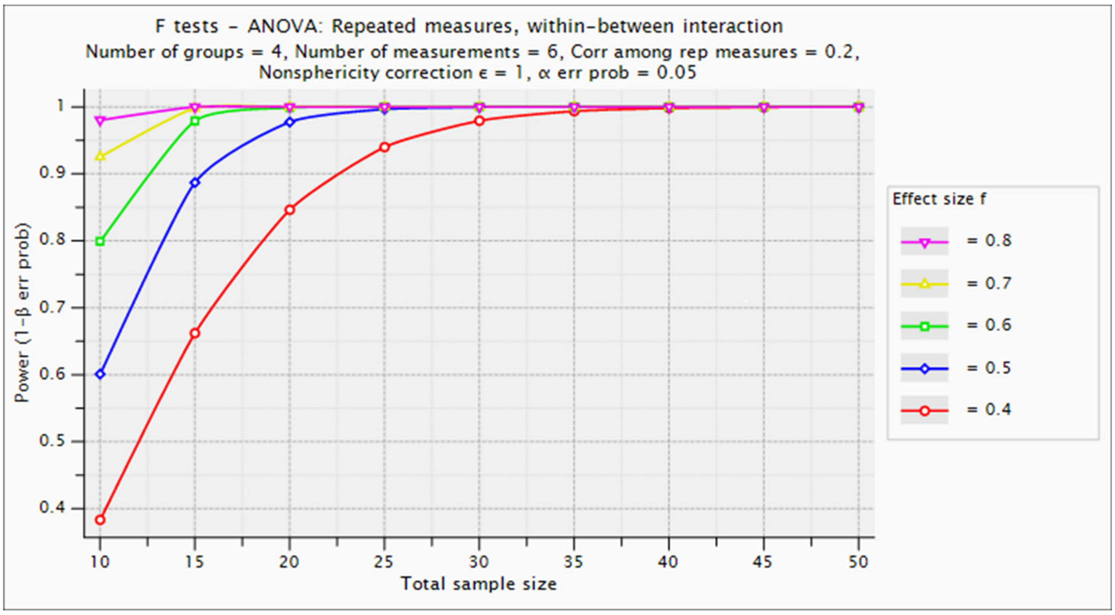

B) BW, Age at slaughter, ADG, Mortality

F tests - ANOVA: Repeated measures, within-between interaction

Analysis: A priori: Compute required sample size

Input: Effect size f = 0.5
α err prob = 0.05
Power (1-β err prob) = 0.95
Number of groups = 4
Number of measurements = 4
Corr among rep measures = 0.2
Nonsphericity correction ε = 1

Output: Noncentrality parameter λ = 30.0000000
Critical F = 2.0400981
Numerator df = 9.0000000
Denominator df = 60.0000000
Total sample size = 24
Actual power = 0.9697843

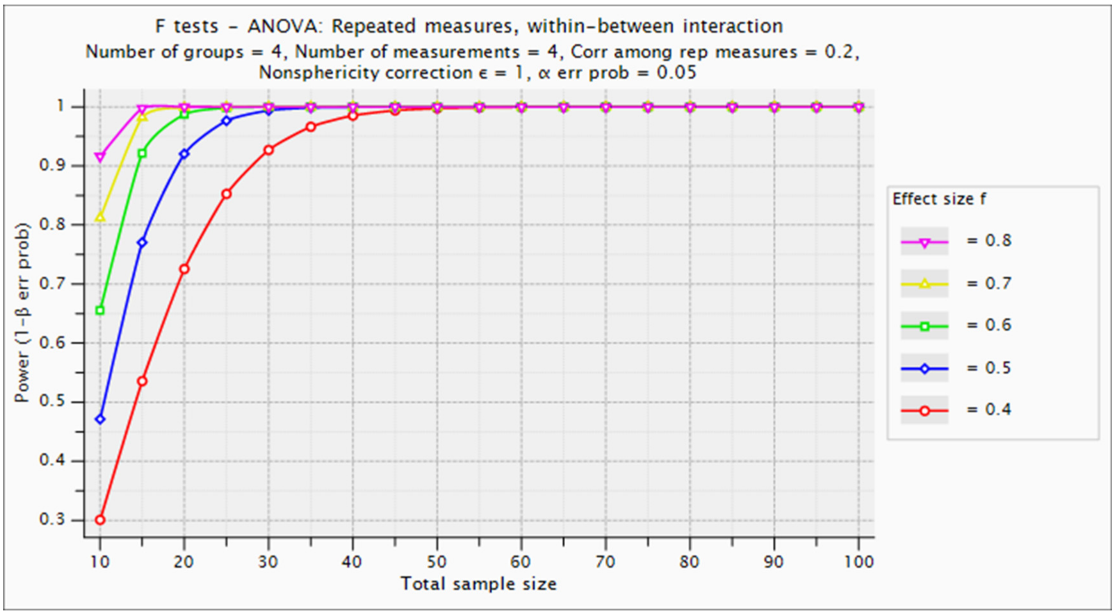

C) Lung score

F tests - ANOVA: Fixed effects, omnibus, one-way

Analysis: A priori: Compute required sample size

Input: Effect size f = 0.75

α err prob = 0.05

Power (1-β err prob) = 0.95

Number of groups = 4

Output: Noncentrality parameter λ = 20.2500000

Critical F = 2.9011196

Numerator df = 3

Denominator df = 32

Total sample size = 36

Actual power = 0.9578945

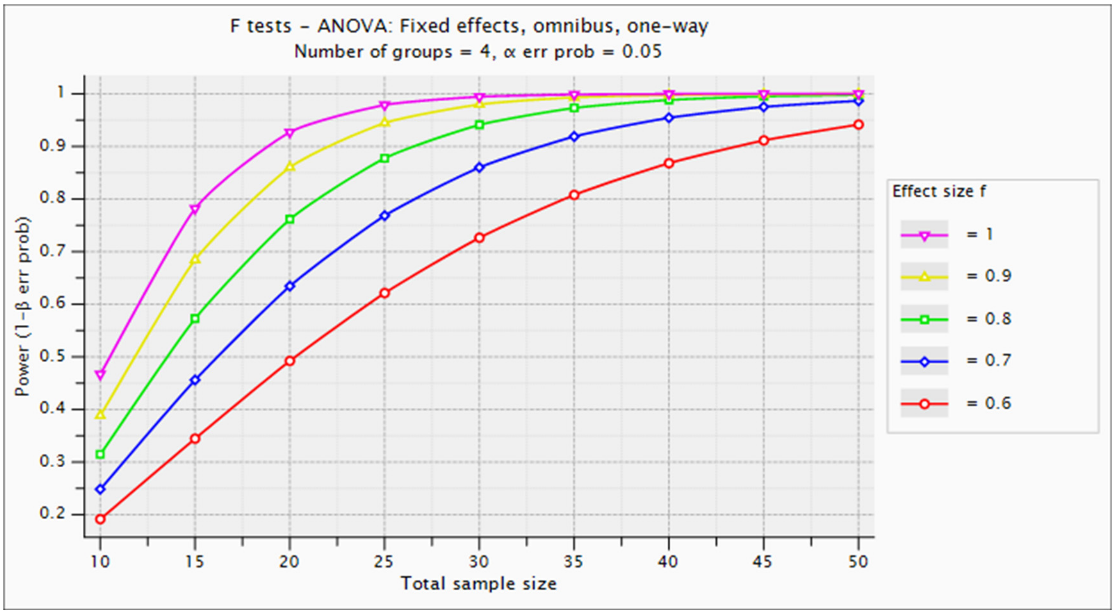

Supplement: Supplementary file 1 [file animals-13-00061-s001.zip › animals-2055929-Supplementary File S1.pdf]
